# SeqSIMLA: a sequence and phenotype simulation tool for complex disease studies

**DOI:** 10.1186/1471-2105-14-199

**Published:** 2013-06-20

**Authors:** Ren-Hua Chung, Chung-Chin Shih

**Affiliations:** 1 Division of Biostatistics and Bioinformatics, Institute of Population Health Sciences, National Health Research Institutes, Zhunan, Miaoli, Taiwan

## Abstract

**Background:**

Association studies based on next-generation sequencing (NGS) technology have become popular, and statistical association tests for NGS data have been developed rapidly. A flexible tool for simulating sequence data in either unrelated case–control or family samples with different disease and quantitative trait models would be useful for evaluating the statistical power for planning a study design and for comparing power among statistical methods based on NGS data.

**Results:**

We developed a simulation tool, SeqSIMLA, which can simulate sequence data with user-specified disease and quantitative trait models. We implemented two disease models, in which the user can flexibly specify the number of disease loci, effect sizes or population attributable risk, disease prevalence, and risk or protective loci. We also implemented a quantitative trait model, in which the user can specify the number of quantitative trait loci (QTL), proportions of variance explained by the QTL, and genetic models. We compiled recombination rates from the HapMap project so that genomic structures similar to the real data can be simulated.

**Conclusions:**

SeqSIMLA can efficiently simulate sequence data with disease or quantitative trait models specified by the user. SeqSIMLA will be very useful for evaluating statistical properties for new study designs and new statistical methods using NGS. SeqSIMLA can be downloaded for free at http://seqsimla.sourceforge.net.

## Background

Computer programs that can simulate genotypes with phenotypes based on user-specified disease or quantitative trait models are essential in genetic studies. They can be used to evaluate statistical power when planning a study design based on the proposed sample size, the assumed genotypic relative risks (GRR), and allele frequencies. They are also useful for evaluating type I error rates for new statistical association tests and power comparisons between the new tests and other existing tests. Therefore, many simulation programs have been developed, mostly aiming to generate genome-wide association study (GWAS) data with dichotomous or quantitative traits [1-5].

Next-generation sequencing (NGS) has become a popular technique for identifying novel rare variants associated with complex diseases [6]. Statistical association tests that can account for rare variants have also been developed rapidly [7-10]. These tests aim to identify multiple rare causal variants in a group of variants selected by biological functions, such as exons, genes, and pathways. A common approach is to pool all the variants in the group to increase the statistical power for associations. To evaluate the statistical power for new tests, a simulation tool that can simulate multiple rare casual variants based on sequence data is necessary. However, simulation programs developed for GWAS may not be appropriate for evaluating statistical properties for NGS studies, because they were designed to simulate common variants based on GWAS panels (e.g., Illumina and Affymetrix) or HapMap project data [11]. Thus, computer software that can simulate sequence data based on realistic models with phenotypes becomes important.

To our knowledge, SimRare is the only existing public software designed specifically to simulate sequence data with phenotypes [12]. SimRare has three modules, including a sequence generation module, a module for phenotype generation based on genotypes, and a module for evaluating association methods. The forward-time simulation algorithm [5,13] is used in SimRare to generate variant data. SimRare focuses on generating unrelated samples and on evaluating association methods developed for unrelated samples. As more and more family-based association studies using NGS are conducted [14-17], software that can generate sequence data in families will be very useful for evaluating the properties of family-based NGS analysis.

We developed the Sequence and phenotype Simulator, SeqSIMLA, which can simulate sequence data in unrelated case–control or family samples with user-specified disease or quantitative trait models. SeqSIMLA uses GENOME [18] as the default sequence generator, which efficiently generates data using the coalescent model. SeqSIMLA also accepts a population of sequences generated by other sequence generators. SeqSIMLA can simulate multiple causal variants in regions on different chromosomes, where the recombination rates between regions are based on the rates estimated from the Hap Map project [11] or a user-specified fixed rate. We compared the features between SeqSIMLA and SimRare and used simulations to demonstrate that SeqSIMLA can generate data in a reasonable time frame.

## Implementation

### Sequence generation

GENOME is used as the default tool to simulate a population of sequences based on the coalescent model. Alternatively, as other sequence simulators can have their own unique features, SeqSIMLA also accepts a population of sequences generated by other programs. GENOME either accepts different recombination rates among chromosomal blocks or assumes a fixed rate across the genome. There is no recombination within each of the chromosomal blocks. To simulate block structures similar to real populations, we downloaded the recombination hotspots across the genome from the HapMap project [11], with the highest recombination rate in each hotspot region used as the recombination rate for the center of the hotspot. Crossovers during meiosis are simulated based on the recombination rates for the centers of hotspots. Alternatively, the user can assume that the recombination rates are uniform across the chromosomes, which is the default setting in GENOME.

### Disease models

We do not have restrictions on the number of disease loci to be simulated. A logistic function as follows is used to calculate the penetrance:

$$P\left(\mathrm{Affected}|X\right)=\exp\left(\alpha +\mathrm{BX}\right)/\left(1+\exp\left(\alpha +\mathrm{BX}\right)\right)$$

where ***X =*** (*G*_*1*_,*G*_*2*_,…,*G*_*n*_) is a vector of genotype coding for *n* disease variants, ***B***= (*β*_1_, *β*_2_, …, *β*_*n*_) is a vector of the conditional log of odds ratios for the associated genotypes, and *α* determines the disease prevalence *K*. The parameter *α* is ln (*f*_0_/(1 − *f*_0_)), which is the log odds of the penetrance for ***X*** with no mutant alleles. The odds ratio *e*^*βi*^ represents the increased odds for the disease for an additional mutant allele at variant *i*[19]. For the prevalence model (Model 1), the disease prevalence *K* is specified by the user. We iteratively search for *α* in the range between −20 and 20 and calculate disease prevalence *K*_*i*_ based on *α*_*i*_ in iteration *i*. The value *α*_*i*_ is selected for *α* if |*K*_*i*_ − *K*| < *ϵ*, where *ϵ* is small (e.g., 0.001). Alternatively, the user can specify *f*_0_ directly, and uses the population attributable risk (PAR) to determine the GRRs for the disease loci (the PAR model or Model 2). The logistic function can be represented by the function of *f*_0_ and GRR :

PAffected|X=f0×∏i=1nGRRik1−f0+f0×∏i=1nGRRik

$$\mathrm{GR}R_{i}=\frac{\mathrm{PA}R_{i}}{\left(1-\mathrm{PA}R_{i}\right)\times R_{i}}+1,$$

where *f*_*0*_ is the baseline penetrance specified by the user, *GRR*_*i*_ is the GRR for the genotype at marker *i*, *PAR*_*i*_ is the population attributable risk, and *R*_*i*_ is the risk allele frequency for marker *i*. The sum of *PAR*_*i*_ for the disease loci is equal to the overall PAR specified by the user. The parameter *k* is coded as the number of mutant allele counts (0, 1, 2) for an additive model, the presence/absence of an mutant allele (2/0) for a dominant model, and the presence/absence of a homozygous mutant genotype (2/0) for a recessive model. The model can assume that rarer variants have higher GRR values, given all causal variants contribute equally to the total PAR. SeqSIMLA can also randomly generate a PAR for each of the disease loci, while keeping the overall PAR fixed. Alternatively, the user can specify a fixed GRR across all disease loci.

The user can simulate dominant, recessive, or additive models for the disease loci under Models 1 and 2. The disease model is determined by the genotype coding in ***X*** for Model 1 and by the parameter *k* for Model 2. For Model 1, the user can specify whether a variant has a risk or protective effect using the parameters in ***B***. For Model 2, the GRR for variant *i* with a protective effect is the inverse of *GRR*_*i*_. The user can also specify the proportion of risk variants in all variants with effects.

### Quantitative trait

We also do not have restrictions on the number of quantitative trait loci (QTL). The user needs to specify the total phenotypic variance *V*_*P*_ and the proportion of variance explained by each of the QTL. Assuming that the proportion of variance explained by QTL *j* is *f*_*j*_ and the allele frequencies for QTL *j* are *p*_*j*_ and *q*_*j*_, the genotypic value *a*_*j*_ can be calculated for additive, dominant, and recessive models as follows [20]:

Additive model: $a_{j}=\sqrt{\frac{V_{P_{j}}}{2p_{j}q_{j}}}$

Dominant model: $a_{j}=\sqrt{\frac{V_{P_{j}}}{8p_{j}q_{j}^{3}+4p_{j}^{2}q_{j}^{2}}}$

Recessive model: $a_{j}=\sqrt{\frac{V_{P_{j}}}{8p_{j}^{3}q_{j}+4p_{j}^{2}q_{j}^{2}}}$

where $V_{P_{j}}=V_{P}f_{j}$.

Assume QTL *j* has two alleles *A*_*1*_ and *A*_*2*_, where *A*_*1*_ is the minor allele responsible for the larger value in the trait. For a set of *M* QTL, the phenotypic value *Y* is a random variable defined as:

$$Y=\mu +\sum_{\left(j=1\right)}^{M}G_{j}+P+E,$$

where *μ* is the general mean specified by the user, *G*_*j*_ follows a normal distribution with mean *μ*_*j*_ and variance *V*_*Pj*_, *P* follows a normal distribution with mean 0 and variance *V*_*poly*_ specified by the user, and *E* follows a normal distribution with mean 0 and variance $\left(V_{P}-\overset{M}{\underset{j=1}{\sum }}V_{P_{j}}-V_{\mathrm{poly}}\right)$. *P* and *E* model the polygenic and environmental components, respectively. The mean *μ*_*j*_ for *G*_*j*_ is defined as:

$$\mu_{j}=\left\{\begin{array}{c} a_{j},0,-a_{j}\;\mathrm{for}\;\mathrm{genotypes}\;A_{1}A_{1},A_{1}A_{2},A_{2}A_{2}\\ \;\mathrm{under}\;\mathrm{the}\;\mathrm{additive}\;\mathrm{model}\\ a_{j},a_{j},-a_{j}\;\mathrm{for}\;\mathrm{genotypes}\;A_{1}A_{1},A_{1}A_{2},A_{2}A_{2}\\ \;\mathrm{under}\;\mathrm{the}\;\mathrm{dominant}\;\mathrm{model}\\ a_{j},-a_{j},-a_{j}\;\mathrm{for}\;\mathrm{genotypes}\;A_{1}A_{1},A_{1}A_{2},A_{2}A_{2}\\ \;\mathrm{under}\;\mathrm{the}\;\mathrm{recessive}\;\mathrm{model} \end{array}\right)$$

### Data types

**Figure 1:**
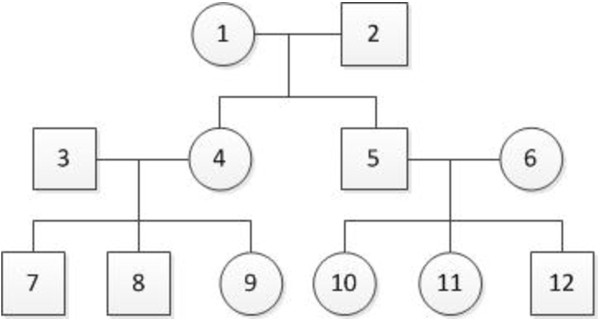
Family structure simulated in SeqSIMLA.

SeqSIMLA can simulate two data types – three-generation family data with 12 members and unrelated cases and controls. The structure for each family is shown in Figure 1. We assume random mating in a population of haplotypes generated by GENOME to simulate family data. For the disease models, a family is ascertained if there is a user-specified number of affected siblings (e.g., 1–3) in the third generation. To generate case–control data, we simulate cases by randomly selecting unrelated affected individuals and simulate controls by randomly selecting unrelated unaffected individuals in the third generations of unrelated families. For the quantitative trait model, the user can decide whether the families will be ascertained based on affection status in family members, which is the same procedure as in the disease models, or randomly from the population.

### Efficiency improvements

SeqSIMLA determines that an individual is affected by comparing the probability calculated from the penetrance function given the person’s genotypes to a random number. This process can be inefficient for a rare disease with low penetrance. We implemented a similar strategy as in Edwards et al. [21] to efficiently simulate unrelated cases. We first simulated a small set of cases (e.g. 100 cases) using the penetrance function in Model 1 or 2. The conditional probability P(***X***|Affected) can be calculated based on the set of cases, where ***X*** is a multilocus genotype observed at the disease variants in the set of cases. Then a multilocus genotype at the disease variants for a case is simulated based on the conditional distribution, and two sequence haplotypes, which are consistent with the multilocus genotype, are randomly selected from the population of sequences. The advantage of this method is that the run time is not affected by the disease prevalence. However, the conditional probability is subject to sampling error as it is estimated in a small set of samples.

As generating each of the families, unrelated cases, and unrelated controls is an independent process, the procedures can be parallelized with threads on a multi-core computer. We used Java Thread to parallelize the code. Each thread generates about the same amount of families, cases, and controls. We used simulations to evaluate the performance of SeqSIMLA running with threads.

## Results and discussion

SeqSIMLA is implemented in Java, which is portable on different operating systems, including Linux and Windows. The parameters required for SeqSIMLA can be specified in the command line. Alternatively, the user can specify the parameters using a control file so that they can be saved and reused. SeqSIMLA writes the variant data in standard PLINK file format (map and ped files) [22], which has been widely adopted in genetic analysis. Map distance between two variants is calculated by Haldane’s mapping function [23]. An additional phenotype file with quantitative trait values, which is also in the same format as the PLINK phenotype file, is generated if the user chooses to simulate a quantitative trait.

We evaluated the performance of SeqSIMLA for generating datasets. We used the parameters provided in the best-fitting population-genetics model [24], which includes estimates of ancestral population sizes, duration of population expansion, migration rates, recombination rates, final population sizes, and gene conversion rate, to simulate a population of sequences with an allele frequency spectrum similar to the European population in GENOME. We simulated 1 block and 50 adjacent blocks on chromosome 1, based on the recombination rates from the HapMap project. As more than 30 functional variants in coding regions can be identified in resequencing studies for complex diseases [25,26], we simulated 30 disease loci with minor allele frequencies less than 0.01 using Model 2. The overall PAR was set as 0.1. The baseline penetrance *f*_*0*_ was set as 0.1, 0.01, and 0.001, reflecting prevalence of different complex diseases. We also simulated two different types of study samples: 500 families with at least one affected sib, and 1000 cases and 1000 controls. The average time (over 100 replicates) spent on generating a dataset was shown in Table 1. The simulations were performed on a Linux server with Intel Xeon 2.4 GHz CPUs (24 cores) and 96 GB of memory. As shown in Table 1, SeqSIMLA can generate a dataset in a 1 block region with 1,177 SNPs in 8 seconds with 12 threads. Even with a rare disease (prevalence 0.0014), SeqSIMLA with threads can generate a dataset in a larger region (50 blocks with 55,681 SNPs) in 5 minutes.

**Table 1:** Run time of SeqSIMLA for generating family and case–control data

	**1 Block**			**50 Blocks**		
	**(1,177 SNPs)**			**(55,681 SNPs)**		
Prevalence^1^	0.11	0.012	0.0014	0.11	0.012	0.0014
Family^2^	4.88 s	6.09 s	16.6 s	3.85 m	4.06 m	7.58 m
	(94%)^7^	(76%)	(30%)	(82%)^8^	(76%)	(46%)
Family (T)^3^	4.58 s	5.33 s	7.52 s	3.35 m	3.45 m	4.51 m
	(98%)	(91%)	(57%)	(87%)	(84%)	(67%)
Case-control^4^	3.17 s	4.86 s	26.35 s	3.10 m	3.75 m	11.01 m
	(64%)	(33%)	(6%)	(34%)	(29%)	(11%)
Case–control (T)^5^	2.02 s	2.57 s	7.56 s	1.96 m	2.76 m	3.68 m
	(88%)	(74%)	(20%)	(34%)	(37%)	(22%)
Case–control (F)^6^	2.42 s	2.44 s	2.27 s	1.95 m	1.87 m	1.87 m
	(91%)	(91%)	(92%)	(58%)	(58%)	(58%)

^1^The estimated prevalence based on 10,000 prospective cohorts generated under Model 2.

^2^500 families generated with 1 thread.

^3^500 families generated with 12 threads.

^4^1000 cases and 1000 controls generated with 1 thread.

^5^1000 cases and 1000 controls generated with 12 threads.

^6^1000 cases and 1000 controls generated with the conditional probability of multilocus genotypes given the disease status.

^7^Run time (seconds) and the percentage of run time spent on I/O.

^8^Run time (minutes) and the percentage of run time spent on I/O.

We performed linkage and rare-variant association analysis on the data simulated by SeqSIMLA to verify that SeqSIMLA is properly implemented. For linkage analysis, 300 families with at least two affected siblings were simulated. We simulated two regions (REGION1 and REGION2) on the same chromosome, where the recombination fraction between the two regions was 0.2. We simulated 5 disease loci using Model 1 with MAF between 0.05 and 0.15 in REGION1, which has 374 variants. The disease prevalence was 0.1. All disease loci were assumed to have an effect size of 1.5, which was commonly observed in GWAS studies [27]. REGION2 has 375 variants. For association analysis, we simulated 1000 cases and 1000 controls. We also simulated two regions (REGION3 and REGION4) on the same chromosome, where the recombination fraction between the two regions was 1. We simulated 30 disease loci using Model 2 with MAF < 0.01 in REGION3, which has 329 variants. The baseline penetrance *f*_*0*_ and the population attributable risk (PAR) were specified as 0.1. REGION4 has 355 variants. A total of 1000 replicates of family and case–control data were simulated for linkage and association analysis, respectively. MERLIN [28] was used to perform linkage analysis (with the --pairs option); and the Sequence Kernel Association Test (SKAT) [29] implemented in the SKAT R package was used to perform association analysis. Table 2 shows the results of type I error rates and power calculated for the null and alternative models, respectively. As shown in Table 2, both MERLIN and SKAT have power for REGION1 and REGION3, where the disease loci are located. MERLIN also has power for REGION2, which is linked to REGION1. SKAT has the correct type I error rate for REGION4, which is not linked to the disease loci.

**Table 2:** Power and type I error rates for linkage and association tests

**Regions**	**Test**	**Rate**^**1**^
REGION1	MERLIN	0.833
REGION2	MERLIN	0.806
REGION3	SKAT	0.948
REGION4	SKAT	0.040

^1^The proportion of tests being rejected at the 0.05 significance level over 1000 replicates.

Two penetrance functions, Models 1 and 2, are used to determine disease status in SeqSIMLA. Model 1, which is based on the logistic function and has been used extensively in many simulation studies [19,30,31], allows the user to determine the conditional odds ratio for each of the disease variants and the disease prevalence. Therefore, the user can simulate disease models based on estimated odds ratios of candidate variants from previous association studies and estimated disease prevalence from a prevalence study. Model 2, which is based on the population attributable risk, has the advantage of controlling the overall PAR for a group of disease variants. The model can assume that rarer disease variants have higher GRRs, given that all of the variants have the same PAR [8]. The model can also assume disease variants contribute unequally to the overall PAR. Therefore, the model is suitable to simulate a large number of rare disease variants with different odds ratios, while keeping the overall PAR in a specified value.

Both SeqSIMLA and SimRare are able to generate sequence data for independent samples, but with some different underlying settings. SimRare uses the forward-time simulation program srv implemented in the SimuPOP environment to generate sequence data. The srv program provides multi-locus selection models with random fitness effects, which are ideal for simulating multiple rare variants under selection. The default sequence generator, GENOME, in SeqSIMLA is a backward-time simulator. Similar to the limitation in other backward-time simulators, selection is not modeled in GENOME [32]. However, the backward-time simulators are generally faster than the forward-time simulators. For simulating disease status, both SeqSIMLA and SimRare allow the user to specify the odds ratios or population attributable risk for disease variants, the proportion of protective variants, the mode of inheritance, and the disease prevalence. For simulating quantitative trait values, SeqSIMLA allows the user to specify the total phenotypic variance, the proportion of variance explained by each of the causal variants, and the mean of the trait values, while SimRare allows the user to specify the deviations from the mean.

Table 3 shows the comparisons of features between SeqSIMLA and SimRare. Both tools provide multiple disease and quantitative trait models with flexible parameter settings. However, SeqSIMLA has two major advantages over SimRare. First, SeqSIMLA can simulate three-generation families in addition to case–control data, while SimRare simulates only case–control samples. Therefore, SeqSIMLA will be very useful for studying the statistical properties for family-based design. Second, SeqSIMLA is able to simulate different recombination rates between chromosomal blocks, while SimRare assumes a fixed recombination rate. This feature in SeqSIMLA will enable the user to simulate different linkage disequilibrium (LD) structures among chromosomal blocks. On the other hand, SimRare has some unique properties that can be potentially implemented in SeqSIMLA. For example, the power comparison module allows the user to perform a power study based on existing and newly developed statistical tests, and the graphical user interface provides a user friendly interface for parameter settings.

**Table 3:** Comparisons of features between SeqSIMLA and SIMRARE

**Feature**	**SeqSIMLA**	**SimRare**
Language	C++/Java	Python/C++
Sequence simulator	GENOME (default) or a sequence pool generated by a simulator	srv in SimuPOP
Simulated region	Multiple genes on multiple chromosomes	A gene or multiple independent genes
Simulated data type	Families/unrelated cases and controls	Unrelated cases and controls
Recombination rate	Variable/Fixed	Fixed
Number of disease/trait models	3	6
User interface	Command line	Graphical

## Conclusions

We implemented two disease models in SeqSIMLA, in which the user can flexibly specify the number of disease loci, effect sizes or PAR, disease prevalence, and risk or protective loci. We also implemented a quantitative trait model, in which the user can specify the number of QTL, proportions of variance explained by the QTL, and genetic models. We compiled recombination rates from the HapMap project, so that genomic structures similar to real data can be simulated. Future development of SeqSIMLA includes more flexibility in simulating family structures, such as twins or multi-generation families. SeqSIMLA can be used as a complementary tool to SimRare. If the user would like to perform a power study based on case–control design for new and existing statistical methods, SimRare is ideal. If the user would like to perform a power study for family-based design, or to simulate causal variants in multiple genes with different LD patterns among genes, SeqSIMLA is more suitable. In summary, as statistical methods for rare variant association analysis are developing rapidly, SeqSIMLA will be useful for evaluating statistical properties for the new methods based on case–control or family designs. SeqSIMLA will also be useful for power studies when planning association studies based on NGS.

## Availability and requirements

**Project name:** SeqSIMLA

**Project home page:**http://seqsimla.sourceforge.net

**Operating system(s):** Unix, Linux, Windows

**Programming language:** Java

**Other requirements:** Java JDK 7

**License:** GNU GPL

**Any restrictions to use by non-academics:** None

## Competing interests

The authors declare that they have no competing interests.

## Authors’ contributions

RHC was the primary author on the manuscript and designed the algorithm of the program. CCS contributed to the implementation of the software and discussions of the algorithm. Both authors read and approved the final manuscript.
